# Screening of Anal HPV Precancerous Lesions: A Review after Last Recommendations

**DOI:** 10.3390/jcm13175246

**Published:** 2024-09-04

**Authors:** Alessio Natale, Tullio Brunetti, Gionathan Orioni, Valeria Gaspari

**Affiliations:** 1 Dermatology Unit, IRCCS Azienda Ospedaliero-Universitaria di Bologna, Policlinico S. Orsola-Malpighi, 40138 Bologna, Italy; 2 Department of Medical and Surgical Sciences, Alma Mater Studiorum University of Bologna, 40126 Bologna, Italy

**Keywords:** HPV anal cancer, HPV precancerous lesions, screening, papillomavirus

## Abstract

Over the last decades, the incidence of anal cancer has increased worldwide. The discovery of the HPV virus as its primary cause and the natural progression of the disease, involving precancerous lesions, have resulted in significant interest in screening for anal cancer. The use of cytology testing, high-risk HPV DNA research, high-resolution anoscopy, and their combination has been adopted with variable success in detecting anal HPV precancerous lesions. Various studies have been carried out to evaluate the sensitivity and specificity of these techniques in different populations. High-risk populations for developing anal cancer have been identified through study of incidence and prevalence. Therefore, different scientific societies and experts worldwide have provided different recommendations for screening, but a universal approach has not yet been established. The inhomogeneity of different risk groups, the variable accessibility to specifical techniques, and the lack of data regarding the cost–benefit ratio of screening are the main problems to address in order to define a consensus guideline acceptable worldwide. The purpose of this paper is to provide a comprehensive review of the literature on HPV precancerous lesions and its screening, particularly after the release of recent recommendations.

## 1. Introduction

Human papillomavirus (HPV) is a small, double-stranded, non-enveloped DNA virus that leads to epithelial proliferation on both mucosal and cutaneous surfaces [1]. HPV represents the most common etiologic agent of sexually transmitted infections (STIs) [2], but it is also reputed to be the primary cause of anal carcinoma [2]. HPV 16, 18, 31, 33, and 45 are considered subtypes that pose a high risk of developing cancer. In particular, HPV16 (75–80%) and HPV18 (about 3.5%) are the most prevalent genotypes isolated in anal cancers [3,4,5]. Frequently, this virus causes transient and asymptomatic infections that are quickly cleared by an intact immune system. In other cases, a high-risk HPV subtype infection can lead to the development of malignant lesions, such as anal cancer. 

Anal cancer accounts for approximately 2% of the malignancies in the gastrointestinal tract [6]. Between all anal cancers, the most prevalent histologic type is squamous cell cancer (SCC), which represents about 80% of all cases. SCCs are preceded by precancerous lesions, known as squamous intraepithelial lesions (SILs), also known as anal intraepithelial neoplasias (AIN). AIN are graded by how much of the epithelial layer is affected from dysplasia into AIN-1 (low-grade dysplasia) and AIN-II and AIN-III (high-grade dysplasia) [7]. Following the Lower Anogenital Squamous Terminology (LAST) classification, the two types of squamous intraepithelial lesions are the low-grade SIL (LSIL), comprising AIN-1, and high-grade SIL (HSIL), comprising AIN-2 and AIN-3 [8]. According to various studies, the risk of progression from AIN to cancer is higher in people living with HIV (PLHIV) and individuals with a history of HSIL. A study showed a progression from AIN I to AIN III in 12.6% of male high-risk patients, enhancing how PLHIV were more supposed to develop HSIL (hazard ratio: 2.8) [9]. A similar study reported a progression in 19.6% of individuals who had a history of HSIL [10]. According to the same study, progression rates in PLHIV were reduced to 2.8/100 person-years if they were on anti-retroviral therapy or in a stable relationship [10].

The incidence of anal cancer ranges from 0.7 to 1.7 per 100,000 people per year [6]. It affects women more frequently than men [11].

In different countries, the incidence of anal cancer has increased over the last four decades, with an average increase of 2.7% each year [11,12,13]. 

The link between anal HPV infection and anal cancer development has led to a growing interest in the burden of anal HPV infection. That’s the reason why different organizations worldwide are developing screening guidelines for anal cancer prevention. The guidelines differ regarding who should be screened, at what age to initiate screening, the preferred screening tests, and the management of abnormal results.

## 2. Objectives

The primary objective of this paper is to provide the most up-to-date advice for screening for anal HPV precancerous lesions and to give the reader a comprehensive overview of the current state of knowledge on screening techniques and proposed screening recommendations. Furthermore, the paper offers insight into future biomarkers and directions of anal HPV screening for precancerous lesions.

## 3. Methods

**Figure 1:**
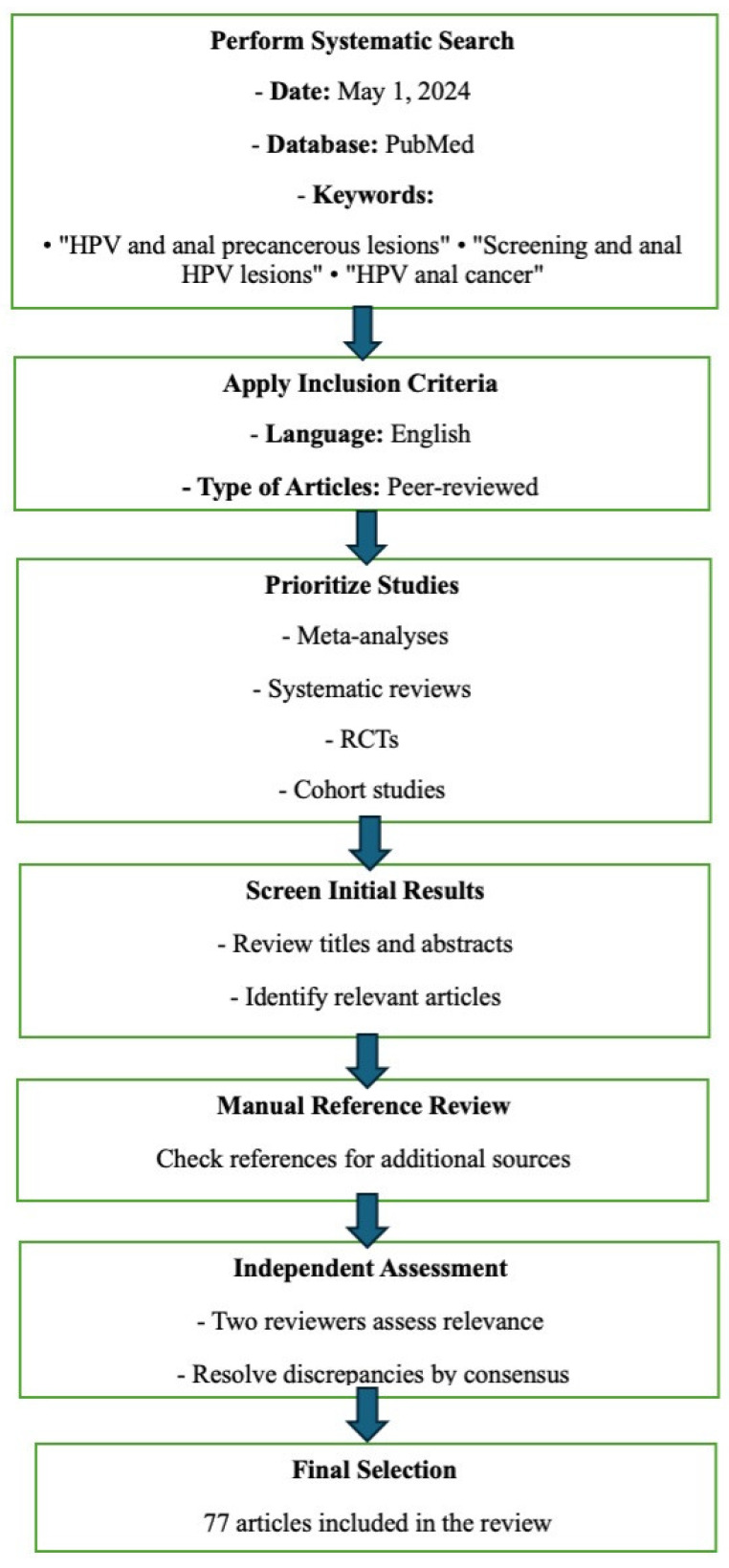
Study flow chart representing the review process performed.

To conduct a comprehensive review of the literature, we performed a systematic search of PubMed on 1 May 2024. A combination of specific keywords was used to capture relevant articles, focusing on the terms “HPV and anal precancerous lesions”, “screening and anal HPV lesions”, and “HPV anal cancer”. We considered only studies published in English and peer-reviewed articles. Preference was given to studies with higher levels of evidence, such as meta-analyses, systematic reviews, randomized controlled trials (RCTs), and cohort studies. The initial search results were screened based on titles and abstracts to identify potentially relevant articles. References to articles were manually reviewed to identify additional sources. Two independent reviewers assessed the relevance of each study. Discrepancies were resolved through discussion and consensus. At the end of this process, only 77 published articles were included in this review (Figure 1).

## 4. Relevant Sections

The rationale for screening HPV-related precancerous anal lesions arises from different considerations: the similarities between the anus and cervix, the success of cervical cytology screening in detecting precancerous conditions, and the advantage of treating HSIL in decreasing the progression rate of invasive anal cancer [14].

Screening for HPV-related precancerous anal lesions is possible due to the following factors: the identification of individuals with high risk for developing anal cancer, the availability of screening techniques that are capable of accurately diagnosing the HSIL lesion that precedes anal cancer, the accessibility to effective treatments that can ablate HSIL, and the significant reduction in morbidity and mortality related to anal cancer with early identification and treatment of SIL: in a 25-month follow-up study conducted by the ANCHOR group, treatment of HSIL decreased the risk of progression to SCC by 57% for PLHIV [15].

*High-Risk Populations*

The patient populations that are at highest risk of developing SIL are reported in Table 1.

Studies on anal SIL focus mainly on men who have sex with men (MSM), as they are considered to be more susceptible to developing anal cancer than males who do not have sex with men. Developing anal SIL was more likely to occur when there were more than five sexual partners or five receptive partners for anal intercourse in the previous six months [20]. The same study demonstrated that age does not affect the prevalence of SIL. 

PLHIV are at elevated risk for developing anal HPV-related SIL and invasive anal cancer [16]. While anal cancer incidence in the general population is approximately 1.5 per 100,000 person-years [13], data from the United States registries found an incidence of 50.7 per 100,000 person-years in PLHIV [21]. The same study showed that among men who have sex with men living with HIV (MSMLWH), the incidence per 100,000 person-years was 88.7, while other males with HIV had an incidence of 31.9 and women who live with HIV (WLWH) had 24.2. Low baseline CD4+ cell count and a low CD4+ nadir are both linked to a higher prevalence of SIL [19,22,23,24] and a higher risk of anal cancer in PLHIV [25]. HIV infection is regarded as a significant risk factor for developing anal SIL among females [17,18]. The study conducted by Hessol et al. found a prevalence of anal HSIL much higher in WLWH than HIV-negative females (9 versus 1 percent). In another study including a cohort of 256 WLWH, the prevalence of anal HSIL was 27% [26]. How HIV infection impacts the development of anal cancer is still unclear. There are multiple factors that may contribute to the high prevalence of anal SIL and the high incidence of anal cancer in PLHIV, for example the more-likely tendency to have unprotected sex, a higher chance of getting infected with multiple HPV types, and an impaired mucosal immune response that facilitates HPV replication [19,20]. 

A history of cervical HPV infection is an additional risk factor [27,28,29]. In the systematic review conducted by Lin et al., it was found that 25% of females older than 45 with cervical HPV-16 infection had HPV-16-positive anal HSIL [30]. 

Other risk factors for anal SIL in females include a history of anal receptive intercourse [27,28] and the presence of multiple partners [27].

Chronic immunosuppression has been associated with the development of HSIL and invasive anal carcinoma. A meta-analysis about anal cancer incidence by risk group evidenced an incidence rate of 13 cases per 100,000 person-years among solid organ transplant recipients, that was even higher by 10 years post-transplant (24.5 in males, 49.6 for females) [31]. According to other studies, the risk of anogenital cancer can go up to 100 times among kidney transplant recipients [32,33]. In other studies, autoimmune diseases have been found to be a risk factor for anal cancer, and this may be due to the treatment of chronic glucocorticoids [34,35]. 

Finally, an increased risk of anal cancer has been noted in smokers, especially current smokers [25,36].

Screening Tests For Anal Hpv-Related Precancerous Lesions

## 5. Proctological Examination

If a patient exhibits proctological symptoms, the proctological examination (PE) should be performed, in accordance with the recommendations for screening HPV-lesions published by the International Anal Neoplasia Society (IANS) in 2012 [37]. The process involves anal margin inspection, digital anorectal examination, and anoscopy. Although the PE has not been extensively investigated, the procedure is considered to be well-tolerated and acceptable [38,39,40]. Identifying macroscopically visible SCC is possible with this examination. However, anal HSIL is typically not palpable and is unlikely to be discovered during a standard digital anorectal examination [39]. A digital anorectal examination was unable to detect any of the 156 cases of HSIL in a prospective study of 446 men with PLHIV [41]. A study conducted on 1206 PLHIV reported the histological results of anal lesions detected with PE: anal lesions were found clinically in 26% of patients; of these, 9.2% had a lesion with no dysplasia, 10.2% had an LSIL, 6% had an HSIL, and 0.6% had anal cancer [39].

In another study, 212 patients with PLHIV underwent a comparison of screening strategies. The result showed that a combination of PE, anal smear, and a high-risk HPV (HR-HPV) test had a better detection rate in HSIL diagnosis than PE alone, showing up at 12.7% versus 3.3% [42]. 

## 6. Anal Cytology

Anal smear testing involves collecting transitional cells by inserting a water-moistened polyester fiber swab into the anal canal without any preparation or disinfection [43,44]. To prevent loss of cellular yield and morphology related to lubrication, it is important to collect anal cytology before performing digital anorectal examination or anoscopy. The swab is slowly inserted until it touches the rectum wall, and then it is placed proximal to the anorectal transformation zone [19].

It is recommended to perform the smear at least 48 h after the last sexual intercourse, in the absence of any infection or treatment [43,44]. 

An alternative to clinician-collected samples is anal self-sampling (ASS), which is acceptable, feasible, and performs similarly in diagnosis [45,46,47,48]. The assumption is that self-collected samples could improve patient compliance and decrease screening costs, while clinician-collected samples may be slightly more sensitive [49].

The meta-analysis by Clarke et al. revealed that anal cytology was able to detect ASC-US and worst abnormalities with a sensitivity of 81% and a specificity of 62.4% [50], performance that is close to that of cervical smear tests [51]. The sensitivity of anal cytology appears to be higher in MSMLHIV than MSM not living with HIV (85.2% vs. 56.6%, respectively) [50]. According to the SPANC study, the specificity of cytology testing rises with age, and its sensitivity rises with the size and count of HSILs [52]. 

The main limitation of cytology is its lack of specificity, which can result in unnecessary HRA. Additionally, the correlation between the grade of cytological abnormalities and the grade of histological abnormalities is not strong [14]. According to Clarke et al.’s meta-analysis, there was a 35% chance of HSIL in cases of ASC-US+ abnormalities, highlighting how the detection of low-grade cytological abnormalities cannot rule out the presence of HSILs [50]. On the other hand, high-grade cytological abnormalities are highly predictive of HSIL, with a specificity of 96.44% and a sensitivity of 21.1% [50]. 

## 7. High Risk (HR)—HPV DNA Test

During HR-HPV screening, the viral genome of high-risk HPV is detected using PCR on cells extracted from the anal transitional zone [53,54]. 

In Clarke et al.’s meta-analysis, the HR-HPV test showed a sensitivity of 90% for detecting HSILs, with a specificity of 47% in women and 35% in MSMLHIV [50]. In the case of a negative HR-HPV test, the probability of HSIL was estimated to be 4% by the same study [50]. The specificity could be increased by restricting testing to HPV16 (rather than all HR-HPVs), with a consequent decreasing of sensitivity [50,55]. 

In comparing cytology and HR-HPV testing, another study reported similar sensitivities for HSIL detection, with a 67% higher specificity for the HR-HPV test than for cytology [56]. 

Based on the recent recommendations released by French experts, the test for HPV16 could be useful to make risk stratification, even for patients at risk [57]. 

## 8. High-Resolution Anoscopy (HRA)

After obtaining an abnormal cytology result, HRA is commonly performed for a diagnostic biopsy. It generally refers to the examination of the anal canal and perianal areas using a colposcope for illumination and magnification. By applying 5% acetic acid locally, abnormal transitional epithelium areas are identified for biopsy [37].

The grade of cytological abnormalities may not always be consistent with that of histological abnormalities. According to the APACHES study conducted in France on 524 MSMLHIV, the smear test classified LSIL in 11.3% of HRA-diagnosed HSIL cases, while it resulted in normal in 3.4% with HSIL determined by HRA [58].

HRA is reported to have sensitivity between 59% and 100% and specificity between 66% and 74% [59]. High-grade cytological abnormalities are a predictor of a high-grade histological lesion [60]. The presence of HSIL cannot be ruled out by normal or low-grade anal cytology. In fact, as showed in the study conducted by Dalla Pria et al., the 11% of HRA-identified HSILs on biopsies corresponded to normal smear test results [61]. According to several studies, HRA is superior to clinical examination for HSIL screening [42,61,62]. Camus et al. proved that 65.7% of HSILs found by HRA were unnoticeable to the naked eye [63].

In certain circumstances, like scarred anatomy, stenosis, inflammation, and a history of radiation therapy, the application of HRA is limited [58]. Furthermore, significant interoperator variability is reported, with detection rates for HSIL that ranged from 5.1% to 31.3% depending on the center where HRA was performed [58]. Some quality criteria have been defined for centers that perform HRA; in particular, it is recommended to perform a minimum of 50 HRAs per year, and the detection rate for HSIL in patients diagnosed with HSIL on a smear in the previous 3 months should be 90% [64].

Additionally, HRA exams may not always be accessible to patients due to the long learning curve required [65].

### 8.1. Cost–Benefit Ratio of Screening

In the context of anal HPV screening, there is still a lack of comprehensive data in the literature regarding the cost–benefit ratio. This means that while the screening may offer potential benefits in detecting early signs of HPV-related conditions, we do not yet have sufficient evidence to determine whether these benefits outweigh the costs involved. However, the use of rapid and low-cost techniques targeted at high-risk groups may help reduce the economic burden associated with the management and treatment of anal cancer cases. By focusing resources on subjects most at risk, these approaches could potentially offer a more cost-effective strategy, even in the absence of definitive data on the overall cost–benefit ratio of anal HPV screening. Further research is needed to evaluate the overall effectiveness and economic impact of such screening programs.

### 8.2. Future Biomarkers

In order to make early diagnoses and enhance treatment outcomes, new biomarkers are being studied to identify HPV in precancerous and cancerous lesions [66].

Regarding the use of HPV E6/E7 messenger RNA (mRNA) as markers for the detection of premalignant lesions and anal cancer, a recent meta-analysis showed a sensitivity of 74.2% and a specificity of 64.3% [50]. The meta-analysis included six studies with high heterogeneity of the results.

The use of p16INK4a (p16) and Ki-67 staining techniques can help distinguish LSIL from HSIL in histological specimens [67]. Due to the lack of data, the effectiveness of these markers in anal cytology remains uncertain [68,69].

Methylation markers (host genome and viral DNA) are being evaluated to identify high-grade anal lesions with various degrees of risk of progression to cancer [70,71,72]. 

In terms of prognostic evaluation, the use of HPV as a biomarker remains controversial in clinical practice [66].

## 9. Discussion

The most recent recommendations about screening of anal HPV precancerous lesions are synthesized in Table 2.

A panel of 40 French experts has recently given their recommendations about the screening of HPV-related anal lesions: PE should be performed if the patient presents proctological symptoms. They propose screening the following groups with an HPV test: MSMLHIV older than 30 years-old, women with a history of precancerous lesions or vulvar cancer, women who underwent solid organ transplants more than 10 years ago. They suggest performing surveillance tests every 5-year interval in the absence of HPV16 infection. HPV testing and cytology should be performed after 3 years when HPV16 infection is present and clinical examination is normal. In the case of a clinical examination of abnormal or cytological abnormalities graded at least ASC-US+, patients should undergo HRA. 

Recently, IANS released consensus guidelines for anal cancer screening [73]. The task force classified individuals in risk categories A and B based on the incidence of anal cancer reported in those specific populations. Risk category A (that should undergo a regular screening program) includes MSM and transgender women living with HIV aged more than 35 years-old, MSM and transgender individuals with more than 45 years, individuals with a history of vulvar HSIL or cancer within 1 year of diagnosis, and recipients of solid organ transplants from 10-years post-transplant. Risk category B (that could be included for screening with shared decision-making, based on the availability of HRA) included individuals above 45 years-old with history of cervical/vaginal HSIL or cancer, or individuals with history of perianal warts, persistent cervical HPV 16 (>1 year), or autoimmune diseases. PE should be performed at all screening visits. Either anal cytology alone or HPV testing alone is acceptable for anal cancer screening (quality of evidence BII). Repeat cytology screening in 12 months is recommended for individuals with negative results for intraepithelial lesions or malignancy (NILM) cytology or individuals testing HPV negative. Cytology and HR-HPV co-testing is acceptable for anal cancer screening (BII). They recommend immediate HRA for individuals with ASC-US+ cytology and HPV positive test results, ASC-H or HSIL cytology, and individuals testing HPV16 positive. Repeat screening in 12 months is recommended for individuals with ASC-US cytology testing HPV negative and in 12–24 months for those with NILM cytology testing HPV negative. In settings with limited HRA capacity, it is acceptable to only refer individuals with high-grade cytology (HSIL) or atypical squamous cells and cannot exclude HSIL (ASC-H) to immediate HRA, with repeat testing in 12 months recommended for individuals with low-grade cytology (LSIL) or ASC-US and repeat testing in 12–24 months with NILM results.

Other recommendations from expert groups are reported below: 

The Centers for Disease Control convened an expert advisory group to establish the 2021 guidelines for sexually transmitted infections, and they recommend screening for anal cancer for PLHIV with age 35 or older [74]. Other risk groups include women with a history of HPV-associated genital cancers, solid organ transplant recipients, HIV-negative MSM, and other immunocompromised individuals without HIV infection. Based on their opinion, patients who are not in an increased risk category should not receive anal cancer screening [74].

In 2013, the HIV Medicine Association of the Infectious Diseases Society of America recommended for screening: MSMLHIV, WLHIV with a history of receptive anal intercourse or abnormal cervical Pap test results, and PLHIV with a history of genital warts [75].

Annual anal cytology is recommended by the Society of Transplantation Infectious Diseases Community of Practices for solid organ transplant recipients, especially those who have a history of receptive anal intercourse and/or CIN [76]. 

In 2018, the American Society of Colon and Rectal Surgeons recommended screening anal HSIL in high-risk populations, such as PLWH, MSM, and females with a history of CIN [77].

## 10. Conclusions and Future Directions

Currently, there is no uniform consensus on screening for anal HPV precancerous lesions worldwide. The recent recommendations and evidence-based data from various studies provide a solid basis for achieving a universal approach to these patients, although adjustments to resource availability in different centers may be necessary. In addition, the cost–benefit ratio of screening for anal HSIL in any of the high-risk populations has not been established. 

Finally, more investigation and randomized clinical trials are necessary to obtain validated results and to achieve the same level of awareness in managing these patients as it is for cervical HPV precancerous lesions.

## Figures and Tables

**Table 1:** The patient populations that are at highest risk of developing anal squamous intraepithelial lesions (SILs), as reported in the literature (PLHIV: people who live with HIV; MSMLWH: men who have sex with men living with HIV; MSWLWH: men who have sex with women living with HIV; WLWH: women who live with HIV).

High-Risk Populations of Developing Sil	Article
People living with HIV	[16,17,18]
Men who have sex with men (MSM), patients who underwent iatrogenic immunosuppression, women with a history of cervical, vulvar, or vaginal precancerous lesions or cancer, women with a history of cervical HPV 16 infection, individuals with a history of anogenital warts	[19]
Current cigarette smoking	[19]
More than five sexual partners or five receptive partners for anal intercourse in the previous six months.	[20]
PLHIV are at elevated risk for developing anal HPV-related SIL and invasive anal cancer	[16]
PLHIV are at elevated risk for developing anal cancer compared to general papulation. In particular: - In the general population the incidence of anal cancer per 100,000 person-years was 1.5. - In PLHIV the incidence of anal cancer per 100,000 person-years was 50.7. - In MSMLWH the incidence of anal cancer per 100,000 person-years was 88.7. - In MSWLWH the incidence of anal cancer per 100,000 person-years was 31.9 - In WLWH the incidence of anal cancer per 100,000 person-years was 24.2.	[21]
Low baseline CD4+ cell count and a low CD4+ nadir in PLHIV	[19,22,23,24,25]
HIV infection among females	[17,18,26]
The more-likely tendency to have unprotected sex, a higher chance of getting infected with multiple HPV types, and an impaired mucosal immune response that facilitates HPV replication	[19,20]
A history of cervical HPV infection.	[27,28,29,30]
Females with a history of anal receptive intercourse and presence of multiple partners	[27,28]
Chronic immunosuppression among organ transplant recipients	[31]
Chronic immunosuppression among kidney transplant recipients	[32,33]
Autoimmune diseases treated with chronic glucocorticoids	[34,35]

**Table 2:** The most recent recommendations about screening of anal HPV precancerous lesions are synthesized (SPE: standard proctological examination; MSMLHIV: men who have sex with men living with HIV; ASC-US+: Atypical Squamous Cells of Undetermined Significance and worst cytological abnormalities (ASC-H, cHSIL); HRA: high-resolution anoscopy; MSM: men who have sex with men; NILM: negative result for intraepithelial lesions or malignancy).

Recommendations	Article
**SPE** should be performed if the patient presents proctological symptoms. **HPV test** should be performed in the following populations: - MSMLHIV older than 30 years old. - Women with a history of precancerous lesions or vulvar cancer. - Women who underwent solid organ transplants more than 10 years ago. Based on the result of HPV test and clinical examination: - If absence of HPV-16 16 and normal clinical examination: surveillance tests every 5-year interval. - If presence of HPV-16 and normal clinical examination: HPV testing and cytology should be performed after 3 years - If abnormal clinical examination or cytological abnormalities graded at least ASC-US+: patients should undergo HRA	[57]
Patients classified into: - Risk category A: should undergo regular screening program. It includes: ° MSM and transgender women living with HIV aged more than 35 years-old ° MSM and transgender individuals with more than 45 years. ° Individuals with an history of vulvar HSIL or cancer within 1 year of diagnosis. ° Recipients of solid organ transplant from 10-years post-transplant. - Risk category B could be included for screening with shared decision-making, based on the availability of HRA. It includes: ° Individuals above 45 years-old with history of cervical/vaginal HSIL or cancer, ° Individuals with history of perianal warts, persistent cervical HPV 16 (>1 year) or autoimmune diseases. **SPE** should be performed at all screening visits. **Anal cytology or HPV test** is acceptable for anal cancer screening: - If NILM cytology or HPV negative test: repeat cytology screening in 12 months - If ASC-US+ cytology and HPV positive test, ASC-H or HSIL cytology, and HPV16 positive test: immediate HRA - If ASC-US cytology HPV negative test: repeat screening in 12 months - If NILM cytology and HPV negative test: repeat screening in 12–24 months. In settings with limited HRA capacity, it is acceptable to only refer individuals with (HSIL) or atypical squamous cells, cannot exclude HSIL (ASC-H) to immediate HRA, with repeat testing in 12 months recommended for individuals with low-grade cytology (LSIL) or ASC-US, and repeat testing in 12–24 months with NILM results.	[73]

## Data Availability

The data that support the findings of this study are available from the corresponding author upon reasonable request.

## Abbreviations

AIN	Anal Intraepithelial Neoplasia
ASC-US	Atypical Squamous Cells of Undetermined Significance
ASC-US+	ASC-US and worst cytological abnormalities (ASC-H, cHSIL)
ASC-H	Atypical squamous cells - cannot exclude HSIL
ASS	Anal Self-Sampling
CIN	Cervical Intraepithelial Neoplasia
LAST	Lower Anogenital Squamous Terminology
LSIL	Low-grade SIL
HPV	Human Papillomavirus
HRA	High-Resolution anoscopy
cHSIL	Cytological High-grade SIL
hHSIL	Histopathological High-grade SIL
PE	Proctological Examination
PLHIV	People Living with HIV
SCC	Squamous Cell Cancer
SIL	squamous intraepithelial lesions
STIs	sexually transmitted infections
WLWH	Women Living with HIV
